# Collision tumor of the colon – colonic adenocarcinoma and ovarian granulosa cell tumor

**DOI:** 10.1186/1477-7819-5-118

**Published:** 2007-10-20

**Authors:** Mayur Brahmania, Chandra S Kanthan, Rani Kanthan

**Affiliations:** 1 Departments of Pathology, Royal University Hospital, 103 Hospital Drive, Saskatoon, SK, Canada; 2 Department of Surgery, Royal University Hospital, 103 Hospital Drive, Saskatoon, SK, Canada

## Abstract

**Background:**

Collision tumors of the colon are rare. We report the first case, to our knowledge in the English literature, of a collision tumor composed of a colonic adenocarcinoma arising in a sigmoid diverticulum coexisting with a recurrent ovarian granulosa cell tumor.

**Case presentation:**

A 64-year old woman presented with small bowel obstruction and a large, heterogenous, solid/cystic serosal based pelvic mass consistent with a gastrointestinal stromal tumor on imaging. Her significant past history 16-years ago included a bilateral salpingo-oophrectomy with hysterectomy. Surgical removal of the mass and pathological examination revealed the presence of a colonic adenocarcinoma arising in a large sigmoid diverticulum coexistent with a second neoplastic tumor phenotype; confirmed to be a delayed recurrent ovarian granulosa cell tumor. Though coexistent, the two tumor phenotypes respected their boundaries with no diffuse intermingling or transition between them. She developed lung metastases from the recurrent ovarian tumor within 6 months and died within a year of follow-up.

**Conclusion:**

Collision tumors of the colon are rare. This is the first case reported of a collision tumor composed of adenocarcinoma colon and recurrent granulosa cell tumor representing an example of two independent tumors in a unique one-on-another collision. Clinical awareness and recognition of such tumors are important as they will dictate appropriate treatment strategies dependent on the individual biological aggressiveness of each of the tumor components. Our report highlights the need for histopathologists, surgeons, and oncologists to be aware of the rare possibility of collisions tumors. As seen in our case, the delayed recurrence of granulosa cell tumor of the ovary sixteen years after the initial presentation was the key determining factor in tumor recurrence, tumor progression, and tumor metastasis within three months, which ultimately lead to accelerated death within a year of clinical presentation. Thus accurate identification and recognition of the second neoplasm is important as prognosis and survival may be determined by this component as seen in our index case.

## Background

A composite tumor is described as a lesion that has different components of a tumor intermingling in a way wherein the two components are difficult to distinguish from each other in many areas [1]. A true "collision" tumor on the other hand represents a coexistence of two adjacent but histologically different malignant neoplasm's occurring in the same organ without histological admixture or an intermediate cell population zone [1]. Such tumors consist of components with different histogenesis and different tumorigenetic pathways representing a mosaic of two concurrent but independent tumors that have "collided" with each other. Thus, "collision" tumors are synchronous morphologically different neighboring neoplasm's that have expanded into each others territory and are occurring side by side in the same organ. Without special or unique clinical features, such tumors are difficult to diagnose preoperatively and pathological identification of the dual components is often the only way to make a correct diagnosis.

The occurrence of collision tumors in the human body is rare and even rarer in the colon. Reported cases include adenoma of the colon and carcinoid [2], adenocarcinoma of the colon and carcinoid [3], adenocarcinoma of the colon and transitional cell carcinoma of the bladder [4], Non-Hodgkin lymphoma and adenocarcinoma of the colon [5-9], and adenocarcinoma of the colon and peritoneal metastasis of a hepatoid variant of yolk sac tumor [10]. Our case represents an example of a true collision tumor composed of two independent tumors occurring in a unique one-on-another pattern: i) sixteen year delayed recurrence of a granulosa cell tumor of the ovary coexisting with ii) a primary colonic adenocarcinoma. To the best of our knowledge, this is the first reported case in the English literature.

## Case presentation

**Figure 1:**
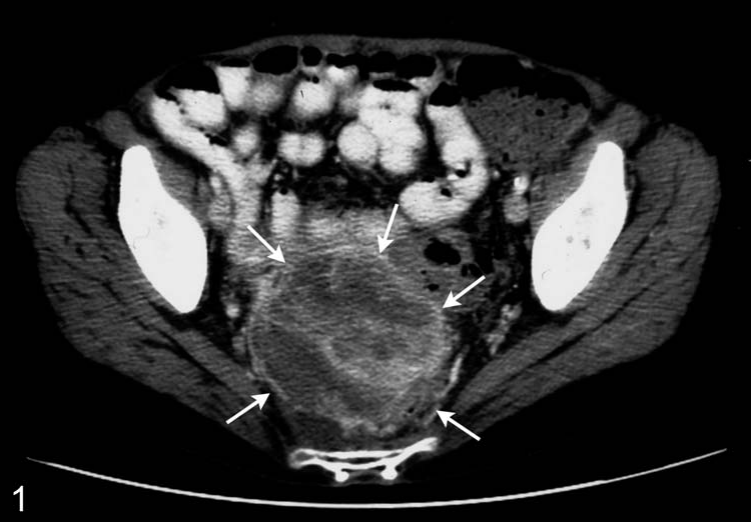
CT scan showing the presence of a large, heterogenous, solid/cystic serosal based pelvic mass (arrows).

A 64 year old woman presented with small bowel obstruction and a pelvic mass. Her significant past history included a bilateral salpingo-oophrectomy with hysterectomy sixteen years ago which on retrospective investigation and tracking of medical records was attributed as surgery for an ovarian granulosa cell tumor. Imaging studies with an abdominal ultrasound showed the presence of a mass which was followed by a CT scan which showed the presence of a large, heterogenous, solid/cystic serosal based pelvic mass that was felt to be an extracolonic gastrointestinal stromal tumor (Figure 1). Positron emission tomography or colonoscopy was not undertaken. Recurrent ovarian carcinoma or a complicated pelvic abscess was also offered as a radiological differential diagnosis. She underwent an explorative laparotomy with *en-bloc *removal of the pelvic mass.

### Pathologic findings

**Figure 2:**
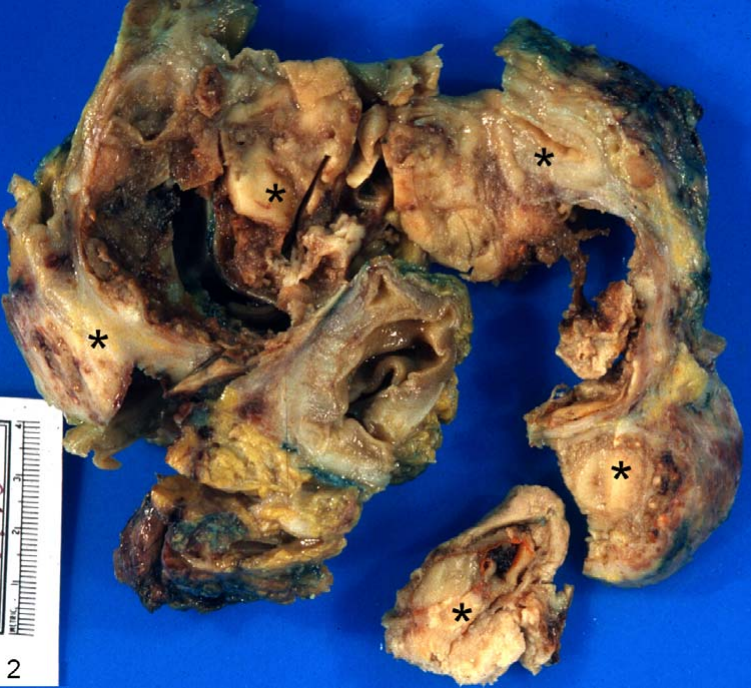
Gross photograph of the specimen composed of colonic segment containing a predominantly serosal based neoplastic lesion measuring 12.0 × 12.0 × 12.0 cm in maximum dimensions (asterisks).

On gross examination, the segment of the colon with overlying fatty tissue and attached firm mass weighed 467 g. The colonic segment measured 25 cm in length and contained a neoplastic lesion measuring 12.0 × 12.0 × 12.0 cm in maximum dimensions. The bowel mucosa was tan colored, unremarkable and showed normal folding pattern overlying the predominantly serosal based neoplastic mass being pale tan in color to red-brown in color with several necrotic areas. Two components of tumor were not identified on gross though there were some streaks of 'orange' colored tissue in one adjacent region of the specimen (Figure 2).

**Figure 3:**
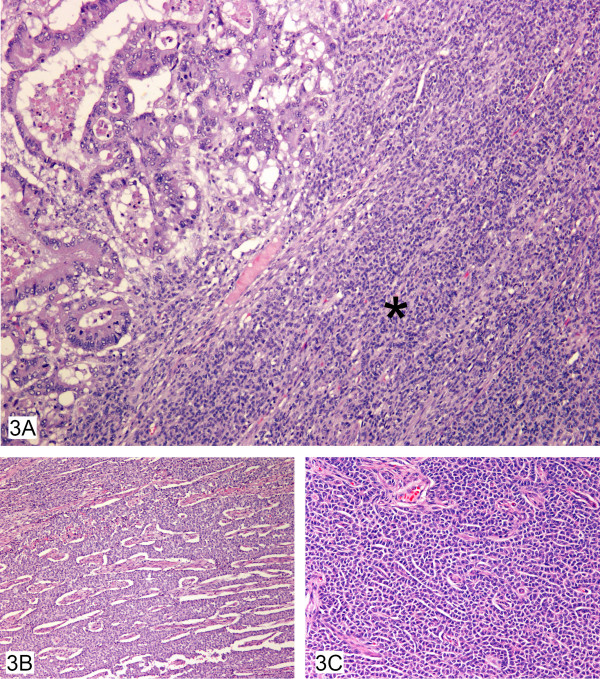
**A, B, C **– Colonic adenocarcinoma colliding with granulosa cell tumor (3A) showing many architectural patterns including diffuse (3A) (asterisk), microfollicular (3B) and cords of cells (3C).

Microscopically, the granulosa cell tumor was composed of fairly large monotonous cells with eosinophilic cytoplasm and nuclei being predominantly oval. Many architectural patterns including diffuse, micro-follicular and cords of neoplastic cells (Figure 3A, B, and 3C) were seen. The recurrence was predominantly seen along the peritoneal surface of the bowel and along the lateral pelvic wall. Isolated adenocarcinoma glandular cells were also identified fortuitously in some regions. Further sections showed the focus of the predominant growth of the typical dirty necrosis with glandular formation of a colonic adenocarcinoma to originate from a diverticular out pouching present within the serosal fat. Detailed sectioning confirmed the connection to the overlying colonic mucosa thus establishing the diverticular status of the bowel. The free "isolated" cells supported the view that the adenocarcinoma had perforated through the diverticulum and was present on the peritoneal surface. These "free tumor cells" though admixed with the granulosa cell tumor cells still respected their individual boundaries. Extensive co-existing diverticular disease of the sigmoid was also present. The adenocarcinoma component was moderately well-differentiated with the mucinous component representing less than 50% of the tumor and there was no evidence of a pre-existing polyp at the site of the carcinoma. The free serosal surface was involved by the ovarian granulosa cell tumor. Sampling of the subserosal fat showed evidence of irregular nodules keeping with venous invasion of an extramural variety along with perineural invasion both from the granulosa cell component. A small focus of giant cell granulomatous response was also observed in the serosal fat. The proximal and surgical margins of the lesion were free of tumor and no separate polyps were identified in the remainder of the bowel sampled. Twenty-one lymph nodes had been harvested from the specimen, of which, one was positive for the presence of metastatic colonic adenocarcinoma.

**Figure 4:**
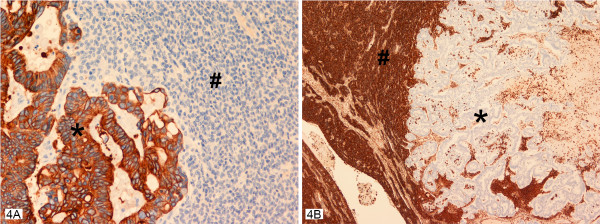
**A, B **– Collision tumor with the two neoplastic components seen with their individual staining pattern with cytokeratin 20 antibodies strongly positive in the adenocarcinomatous component (asterisk) in A and negative in the granulosa cell component (pound). While the vimentin antibodies are strongly positive in the granulosa cell component (asterisk) and negative in the adenocarcinomatous component (asterisk) in B. Inhibin staining pattern was identical to that seen with vimentin antibodies.

Immunohistochemical studies clearly delineated the two components of the collision tumor even in the closely admixed zones on the serosal surface of the lesion. The adenocarcinoma cells were strongly positive to low molecular weight keratin, and CK20 (Figure 4A), while CK7, vimentin, and S100 were negative. This immunohistochemical profile of CK20 being positive and CK7 being negative in the adenocarcinomatous component confirmed the histomorphological diagnosis of colonic adenocarcinoma. P53 antibodies were markedly over expressed in the majority of the adenocarcinoma cells. CA125 remained negative in these cells. Meanwhile, the granulosa cell tumor recurrence regions showed complete negativity to keratin antibodies (low molecular weight keratin, CK 7, CK 20), P53, and CA125. However, these cells were strongly positive to vimentin and inhibin (Figure 4B) with focal positivity with S100 antibodies. This immunohistochemical profile of CK7-, CK20-, inhibin+, vimentin+ confirmed the histomorphological diagnosis of granulosa cell tumor of the ovary. Table 1 summarizes the dilution, clone and the type of the antibodies used in this case.

**Table 1:** Summarizes the different immunohistochemical antibodies used to confirm the two histological components of the collision tumor of the colon.

**Ab/Enzymes**	**Dilution**	**Clone**	**Poly/Monoclonal**	**Animal**
Ck-7	1:35	OVTL12-30	Monoclonal	Mouse
Ck-20	1:25	KS20-8	Monoclonal	Mouse
Low Molecular Weight Keratin	1:50	C51	Monoclonal	Mouse
S100	1:4000	N/A	Polyclonal	Rabbit
Inhibin	1:5	R1	Monoclonal	Mouse
Vimentin	1:4000	V9	Monoclonal	Mouse
CA125	1:20	M11^1^	Monoclonal	Mouse
P53	1:150	DO-7	Monoclonal	Mouse

Ck-7, Inhibin, S100, CA125, p53-DakoCytomation; Mississauga, ON

Vimentin, Low Molecular Weight Keratin-Intermedico; Markham, ON

Ck-20-Novacastra; Norwell, MA

Thus, the final completed pathological analysis of the specimen revealed a primary colonic adenocarcinoma arising in a large sigmoid diverticulum coexisting with another tumor phenotype. The histomorphology and the results of immunohistochemistry, together with the retrieval of the sixteen year old records, confirmed the second neoplasm to be a recurrent ovarian granulosa cell tumor comprising 60% of the specimen. Though coexistent with the primary adenocarcinoma cells, the two tumor phenotypes respected each others boundaries with no diffuse intermingling or transition between the two. At three month postoperative follow-up she developed local recurrence of the granulosa cell tumor in the pelvis and lung metastases within six months confirmed pathologically by image guided fine needle aspiration biopsy. She declined adjuvant chemotherapy and died within a year's follow-up.

## Discussion

Colonic adenocarcinoma is the most common malignant neoplasm occurring in the colon. Collision tumors of the colon on the other hand are extremely rare neoplasms. The admixtures of such two independent tumor phenotypes include the presence of adenocarcinomas with carcinoid [3], transitional cell carcinoma [4], and lymphomas [6-9]. The occurrence of both colonic adenocarcinoma and granulosa cell tumor is uncommon, and to the best of our knowledge has never been reported in the English literature. Although there is no satisfactory explanation for the occurrence of such collision tumors, theories relating to the occurrence of such collision tumors include:

1) Simultaneous proliferation of two different cell lines.

2) Common origin from pluripotent precursor stem cell that differentiates into two components.

3) Chance apposition of two unrelated tumors.

Questions that are not easy to answer and which require further exploration include

a) Are these tumors simple incidental associations?

b) Are these lesions connected by a causal relationship?

c) Does a single carcinogenic agent interact with two neighboring tissues inducing development of tumors of different histological types in the same organ?

Furthermore, in the diagnosis of collision tumors it is important to exclude rare tumors resulting from one cancer metastasizing to one another. In our case, it is hypothesized that some central carcinogenic stimulus induced the development of the primary adenocarcinoma of the colon – a change in the immunological surveillance status – which "awakened" the sleeping dormant granulosa tumor cells resulting in a sixteen year delayed recurrence. Granulosa cell tumors are rarely aggressive, with a five year survival reported to be as high as 86% [11]. The stage of the disease is critical for prognosis, as stages 3 and 4 have a five year survival of only 33.3% [12]. Delayed recurrences of granulosa cell tumors a well known, though uncommon, has a median relapse time of 4–6 years [13] with the longest reported delayed recurrence being 37 years after the initial diagnosis [14]. Further, though colonic adenocarcinoma is a common neoplasm, its origin within a sigmoid diverticulum is uncommon and rare [15-17] adding further complexity to this case in its overall clinical presentation and evolution.

Due to the infrequency of such lesions the biological behavior of colliding tumors is difficult to ascertain in the context of which component will determine the final outcome in terms of disease free survival times. It is debatable whether such outcomes are dependent on either the most predominant component of the collision and/or the more histologically aggressive component of the collision tumor. Molecular genetic analysis may be of special importance for the diagnosis of collision tumors consisting of poorly differentiated neoplasm's such as T cell lymphomas and anaplastic carcinoma when immunohistochemistry remains inconclusive. It is likely such collision tumors are under diagnosed in the routine laboratory.

## Conclusion

Clinical awareness and recognition of such tumors are important as they will dictate appropriate treatment strategies dependent on the individual biological aggressiveness of each of the tumor components. Our report highlights the need for histopathologists, surgeons, and oncologists to be aware of the existence of such rare collisions tumors. As seen in our case, the delayed recurrence of granulosa cell tumor of the ovary sixteen years after the initial presentation was the key determining factor in tumor recurrence, tumor progression, and tumor metastasis within three months, which ultimately lead to accelerated death within a year of clinical presentation. In conclusion, accurate identification and recognition of both components of the collision neoplasm is important in guiding decisions regarding overall prognosis, adjuvant therapeutic options, and survival which may be dependent on either of the components.

## Competing interests

The author(s) declare that they have no competing interests.

## Authors' contributions

SK contributed the surgical aspects of the case, RK contributed the pathological/immunohistochemical portions of the case and conceived of the study, MB conceived of the study, and participated in its design and coordination and helped to draft the manuscript.

All authors have read and approved the final manuscript.
